# Tribochemical Characterization and Tribocorrosive Behavior of CoCrMo Alloys: A Review

**DOI:** 10.3390/ma11010030

**Published:** 2017-12-26

**Authors:** Wei Quan Toh, Xipeng Tan, Ayan Bhowmik, Erjia Liu, Shu Beng Tor

**Affiliations:** 1 Singapore Centre for 3D Printing, School of Mechanical and Aerospace Engineering, Nanyang Technological University, 50 Nanyang Avenue, Singapore 639798, Singapore; tohw0023@e.ntu.edu.sg; 2 Rolls-Royce@NTU Corporate Lab, Nanyang Technological University, 50 Nanyang Avenue, Singapore 639798, Singapore; abhowmik@ntu.edu.sg; 3 School of Mechanical and Aerospace Engineering, Nanyang Technological University, 50 Nanyang Avenue, Singapore 639798, Singapore; MSBTOR@ntu.edu.sg

**Keywords:** tribochemistry, tribocorrosion, micro/nanoscale, additive manufacturing, CoCrMo alloy

## Abstract

Orthopedic implants first started out as an all-metal hip joint replacement. However, poor design and machinability as well as unsatisfactory surface finish subjected the all-metal joint replacement to being superseded by a polyethylene bearing. Continued improvement in manufacturing techniques together with the reality that polyethylene wear debris can cause hazardous reactions in the human body has brought about the revival of metal-on-metal (MOM) hip joints in recent years. This has also led to a relatively new research area that links tribology and corrosion together. This article aims at reviewing the commonly used tribochemical methods adopted in the analysis of tribocorrosion and putting forward some of the models and environmental factors affecting the tribocorrosive behavior of CoCrMo alloys, a widely-used class of biomaterial for orthopedic implants.

## 1. Introduction

The excellent mechanical performance and wear and corrosion resistances coupled with their good biocompatibility have made Co-based alloys, such as CoCrMo alloys, a choice for artificial joints over the last six decades [1,2,3,4]. Once implanted, CoCrMo alloys are subjected to continuing wear and corrosion through body fluid in vivo, which results in the formation of tribological films, wear debris, and metallic ions [5]. If left unaddressed, these wear and corrosion issues associated with the articulating system would pose a serious threat to the long-term safe usage of the artificial joints.

The synergistic action of wear and corrosion resulting in the irreversible transformation of materials in a corrosive environment or aqueous fluid is also known as tribocorrosion [2,6,7,8]. Understanding the fundamental phenomena of tribocorrosion in an articulating system is challenging. Often, these systems are subject to operations under many demanding settings such as sliding, slip, vibration, and weight bearing situations, which further complicate analysis [9]. Given the growing importance of tribocorrosion in bio-related applications, this review aims at highlighting some of the commonly used electrochemical techniques in evaluating the tribochemical behavior of CoCrMo alloys, together with the critical conditions addressed so as to foster a model(s) explaining the phenomena involving both wear and corrosion simultaneously. In addition, some insights on the tribocorrosive behavior of CoCrMo alloys at nano-scale will also be addressed. With the growing interest in the application of nanotechnology to engineering disciplines, such as nanorobotics for diagnosis and targeted drug-delivery, nanoscale tribocorrosion is an extremely relevant discipline and the understanding of which could help pave a way for the realization of electromechanical nanobots based on bacteria models [10,11].

## 2. CoCrMo Alloys and Their Manufacturing Processes

Co-based alloys have been widely used for artificial joints. The most commonly used Co-based alloy in biomedical application is ASTM F75 [1,2,6]. Stellite^TM^ is another name given to the family of Co-based alloys, which is a trademark under Deloro Wear Solutions GmbH, and the comparative series is Stellite^TM^ 21. The elemental compositions of the commonly used Co-based alloys are listed in Table 1. While the alloying contents are in general comparable in the three alloys, a high nickel (Ni) content is seen in Stellite^TM^ 21 [12], which is often added to further improve the corrosion resistance (refer to Section 5.2) of the alloy but is classified as a human carcinogen [13].

Joint implants are often tailored and specific. Therefore, casting has traditionally been offered as the most direct and practical method in producing most of the CoCrMo implants found in the market. However, a coarse carbide structure is often produced resulting in high brittleness and low toughness for the cast CoCrMo alloys [12]. On the contrary, forged CoCrMo alloys that are worked either in the cold or hot environment are often finer and possess equiaxed microstructures, which has rendered forging an effective technique in improving properties as compared to cast alloys [14,15]. Over the last decade, advancement in technologies has allowed CoCrMo implants to be fabricated via additive manufacturing (AM) methods with relative ease. Not only customizable and patient-specific implants can be manufactured with ease, a fine and more dispersed carbide structure can also be achieved through the AM process [16], thus in theory, giving it an improved tribochemical performance.

## 3. Experimental Aspects of Tribocorrosion Study

The study of tribocorrosion phenomena requires knowledge in the areas of both electrochemistry and tribology. Often, the techniques employed in the study of these areas involve well-controlled conditions, which may be difficult to achieve simultaneously in a tribocorrosion experimental set-up. The following section examine some of the mechanical and electrochemical aspects of tribocorrosion experiments with an insight into how the set-up can likewise be applied for nano-scale characterization.

### 3.1. Electrochemical Methods in Tribology

**Figure 1:**
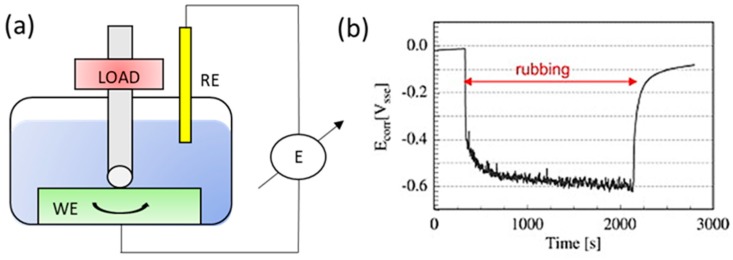
(**a**) Schematic illustration of a typical ball-on-disc (rotating) tribocorrosion experimental set-up that allows the measurement of corrosion potential (RE: standard silver/silver chloride or Ag/AgCl reference electrode); (**b**) Measurement of the corrosion potential of Ti6Al4V alloy rubbing against an alumina ball in a 0.9% NaCl solution (adapted from references [21,22], with permission from © Elsevier), where *E_corr_* = potential difference between working electrode (WE) and RE.

**Figure 2:**
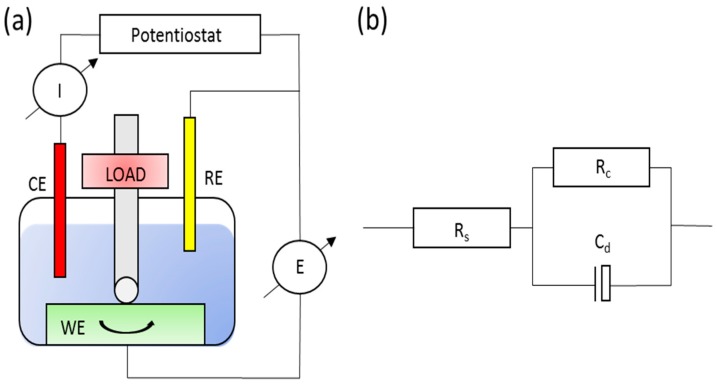
(**a**) A schematic illustration of a typical ball-on-disc (rotating) tribocorrosion experimental set-up that allows for potentiodynamic and potentiostatic measurements; (**b**) a typical Randles circuit used to represent experimental data through electrochemical impedance (EIS) measurement for a tribocorrosion test.

As described in the early pioneering works on tribocorrosion [17,18], tribochemical methods are essentially tribological experiments performed in an anionic conductor under the influence of controlled electrochemical conditions [2,6]. Since the first ASTM G119 Standard Guide for Determining Synergism between Wear and Corrosion was published in 1995, most of the research has focused mainly on identifying the fundamental tribocorrosion mechanisms. However, with the rising adoption of AM technologies and the risks associated with metal-on-polymer (MOP) and ceramic-on-ceramic (COC) bearings [19,20], there has appeared an increase in research focus on the study involving the tribocorrosive behavior of biomedical alloys, particularly CoCrMo alloys. The commonly used electrochemical methods employed for tribocorrosion testing are schematically illustrated in Figure 1 and Figure 2 and discussed thereafter.

#### 3.1.1. Open Circuit Potential (OCP) Measurement

Open circuit potential (OCP) method as schematically shown in Figure 1a is implemented by recording the spontaneous potential difference between the material tested (working electrode, WE) and a reference electrode (RE) immersed in a solution (electrolyte) [21,22]. As illustrated in Figure 1b the corrosion potential shifts to lower values upon the onset of impression and this difference is characterized as the ’cathodic shift’. The measurement of the corrosion potential (*E_corr_*) during wear, mirrors the galvanic coupling between the two surface states of the material involved, namely the exposed metal (oxide removed area) and the passive metal (oxide protected area) [23]. A lower *E_corr_* value below the passivation potential typically directly relates to the situation where the material experiences active dissolution. For a relatively simple, yet easy-to-obtain measurement, the results from OCP measurement offer an insight into the surface state of the material under wear [23,24]. On the contrary, this method provides only limited information in instances where the study of the surface kinetic reaction is involved.

#### 3.1.2. Potentiodynamic Polarization Measurement

Potentiodynamic polarization measurement is essential in defining the active/passive behavior of the materials at varying potentials [21]. The addition of a counter electrode (inert) is required, which is depicted schematically in Figure 2a. Habitually, the measurement is employed to derive the anodic or cathodic current flowing through the working electrode (sample) measured against a reference electrode. Additionally, the effect of friction is also taken into consideration throughout the entire process, which allows observation of its effect on the different electrochemical reactions that occur at different potentials [23].

#### 3.1.3. Potentiostatic Measurement

In this type of measurement, the same physical configuration as in the case of a potentiodynamic test shown in Figure 2a, the current is measured instantaneously at a fixed potential (instead of a varying potential as in the case of the potentiodynamic test) as a function of time for the evolution of the electrochemical kinetics during wear. Essentially, the current measured, *I_measured_*, as described in Equation (1) is the summation of both *I_anodic_* and *I_cathodic_* currents measured across the worn area of the wear track and the surface of the sample not subjected to wear, respectively [21].

(1) $$I_{measured}=I_{anodic}+I_{cathodic}$$

One vital point to note is that during wear, current mainly flows through the worn unpassivated area of the wear track as compared to the overall area of the sample immersed in the solution [24]. Hence, the corresponding current densities (normalized by area) can be several orders of magnitude higher than those measured under a static condition, which further suggests the intensification of metal dissolution.

The method presented is in fact a powerful and valuable technique. The excess current (*I_max_*, maximum current measured during tribocorrosion, *I_o_*, current measured at static condition) can be derived and used to compute the material loss due to corrosion and further provide an avenue for the evaluation of the tribocorrosion model, which is explained in a later section. In addition, various researchers [25,26,27] have developed a model for the repassivation kinetics of the Cr film due to tribocorrosion. Originally proposed by Goldberg et al. [25] and further refined by Sun et al. [28], the exponential decay function can be described as such:

(2) $$I\left(t\right)=I_{o}+C_{1}\exp\left[\frac{-\left(t-t_{o}\right)}{\tau_{1}}\right]+C_{2}\exp\left[\frac{-\left(t-t_{o}\right)}{\tau_{2}}\right]$$

where, *I(t)* is the current level at time *t*, *C*_1_ and *C*_2_ are the experimental current constants and *τ*_1_ and *τ*_2_ are the time constants used to govern the repassivation time.

While *τ*_1_ (proposed by Goldberg et al. [25]) may be sufficient to describe the repassivation kinetics, the introduction of *τ*_2_ (by Sun et al. [28]) was experimentally proven to be more precise as more holistic scenarios (extensively deformed scar area and difficulty of oxygen passivation) were taken into consideration [25,28].

#### 3.1.4. Electrochemical Impedance Measurement (EIS)

A relatively less sought after method in tribocorrosion due to the complexity involved in the heterogeneous state of the material surface, EIS is slowly gaining popularity as it not only enables an in-depth study on the role of intermediate species absorbed on the material surface but also on the properties of the passive film formed. The possibility also extends to the quantification of corroded products and even enables one to simulate an appropriate corrosion condition based on the actual operation requirements by imposing a suitable potential. The concept of EIS involves measuring the current signal of the cell/sample (working electrode) by imposing a small AC potential excitation signal through it. Consequently, the impedance of the target system (*Z*) at time t can be derived as the proportion of imposed electrical potential (*E*) over the response signal (*I*) as [29]:

(3) $$Z=\frac{E}{I}=\frac{E_{o}\sin\left(\omega t\right)}{I_{o}\sin(\omega t+\varphi )}=Z_{o}\;\frac{\sin(\omega t)}{\sin\left(\omega t+\varphi \right)}$$

where *E_o_* and *I_o_* are the amplitude of the original AC potential and the response signal, respectively, ω is the radial frequency, *φ* is the phase shift of the response in a linear system, and *Z_o_* is the resulting magnitude of the impedance due to *E_o_* and *I_o_*. By and large, the impedance signifies a system ability to resist corrosion and a higher *Z_o_* value represents a higher ability to resist corrosion.

Impedance measurements can be performed before, during and after a sliding wear test to assess the effect of wear on the material surface. A modification of the setup is not required and follows that of Figure 2a. The results are often presented in the form of Nyquist diagrams and interpreted by means of an equivalent circuit [30]. A schematic diagram on a typical equivalent circuit used in tribocorrosion studies is depicted in Figure 2b, where *C_d_* represents the charge build-up across the surface (oxide layer or coating), *R_c_* is the resistance due to the material surface layer, and *R_s_* is the resistance of the solution [24,30], when the frequency tends to zero.

**Figure 3:**
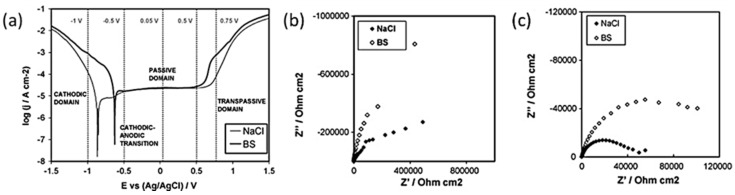
(**a**) Potentiodynamic curves obtained for HC CoCrMo alloy in NaCl and bovine serum [31]; (**b**,**c**) Nyquist diagrams showing the impedance characteristics in NaCl and bovine solution at (**b**) 0.05 *V_Ag/AgCl_* and (**c**) 0.5 V_Ag/AgCl_ (adapted from reference [31], with permission from © Elsevier).

The concept can be further elaborated with the aid of an EIS study done by Igual-Muñoz [31] on high carbon CoCrMo alloy in two different simulated body fluids, namely NaCl and bovine serum. Carefully selected electrical potentials from the potentiodynamic curves need to be in precedence to the EIS test as shown in Figure 3a. These different electrochemical conditions allow for the detailed characterization of metal/electrolyte interface of the CoCrMo alloy, which aids in identifying critical scenarios where metal dissolution is severe. From Table 2, no obvious differences were noticed in the removed wear volume in CoCrMo alloy sliding in both NaCl and bovine serum at applied potentials of 0.05 *V_Ag/AgCl_* and 0.5 *V_Ag/AgCl_*, where generation of passive layer was preferred. However, with the provision of Nyquist diagrams and the corresponding impedance representations as depicted in Figure 3b,c, it could be deduced that dissolution of metal (*U_chemical_*, refer to Section 4.2) was more severe in NaCl than in bovine serum and that this effect was further enhanced at higher passive potential. The higher passive dissolution resistance of CoCrMo alloy in bovine serum is mainly attributed to the release of Co^2+^ ions that result in the enhanced binding of proteins during wear and thus the charge transfer at the metal/solution interface was subsequently contained [31,32].

On the other hand, it was also observed that the contribution of mechanical damage (*U_mechanical_*) decreases slightly at higher passive applied potential in both solutions. This phenomenon was attributed to the formation of thicker passive films where thicker oxide debris particles are more likely to be produced and spalled off during the rubbing process. Consequently, the larger particles have a greater tendency to agglomerate faster compared to the smaller ones, thus forming a tribo-film that protects the underlying surface.

#### 3.1.5. Cathodic Protection or Impressed Current Method

The cathodic protection/impressed current method offers an excellent solution to corrosion control by making the concerned material cathodic instead of anodic in an electrochemical cell [21,33]. The most practical method widely used in industry is to connect the metal to be protected to a more easily corroded metal. Consequently, the concept was extended to tribocorrosion in which Yan et al. [34] used this technique to isolate pure mechanical wear due to biological fluid lubrication and corrosion-related wear arising from the biological fluid. The process involves polarizing the material to make it electro-negative such that the dissolution of the metal ions (immune to corrosion) is not possible. Smooth grooves are predominantly observed under cathodic protection with scanning electron microscopy (SEM), in contrast to the commonly observed pitting and adhesive effect of tribocorrosion [31,35]. The Pourbaix diagram is often utilized in understanding and predicting the material tendency to corrode [36].

### 3.2. Equipment to Study Tribocorrosion

**Figure 4:**
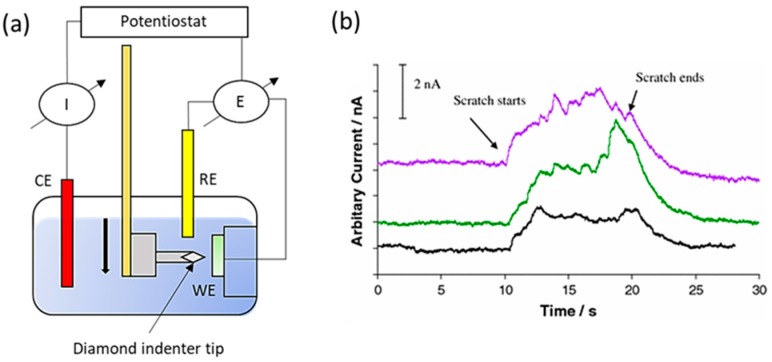
(**a**) A schematic showing the set-up for well-cell nano-scratching experiments; (**b**) Current profiles induced by 3 independent single scratches in NaCl (adapted from reference [37], with permission from © Springer Nature).

Generally, study of tribocorrosion under sliding condition typically uses a cylindrical pin ball or a truncated cone rubbing against a flat plate in an electrolytic solution in a circular or linear reciprocating motion. The set-up is often used in conjunction with the electrochemical method proposed earlier. More recently, Sun et al. [37], extended the study of tribocorrosion to a wet-cell nano-scratching set up where nano-scale tribocorrosion can be performed, as shown in Figure 4a. The corresponding current profiles in Figure 4b, undoubtedly present a trend consistent to the ones due to micro-scratching. An increase in current upon the onset of the scratch (corresponding to the rupture of oxide film) and a current recovery region to the original level at the end of the test are observed. In addition, the scratch current variation was mainly attributed to the effect of the surface morphology such as grain boundaries and carbide distribution [37]. Consequently, a tribologist could extend the concept of nano-scratching and observed in-situ current variation when the scratch passes through a carbide/matrix boundary or even observe the corresponding current characteristics during microstructural change such as strain-induced martensitic ε transformation (SIMT).

It is clear that much of the focus has been on micro-scale tribocorrosion of bulk CoCrMo alloys over the last decade due to the practicability aspect relating to the clinical performance of the alloys. Limited studies involving nano-scale tribocorrosion of CoCrMo alloys have been carried out with emphasis mainly on fundamental aspects of repassivation kinetics and deformation mechanisms. More recently, Martinez-Nogues et al. [38] attempted to carry out some nanoscale characterization on CoCrMo alloy using nanoindentation and reciprocation nano-wear testing under dry conditions. Subsequently, it was shown that under nano-scale wear, a higher load resulted in a lower coefficient of friction (COF), which was contrary to conventional micro-scale wear and was attributed to the reduction in the number of contacting asperities. Consequently, the observation may be of interest when extended to tribocorrosion, especially with the mounting attention being received in the area of nanorobotics.

Nevertheless, it is important to consider two points when dealing with the nano-scale characterization of CoCrMo alloys. One is the typical size of carbide for the selection of indenter size and the other is the dispersion of carbide relative to the selected area for test. An appropriate indenter size will help differentiate the nano-hardness contribution of the metal matrix from that of carbides, thus reducing the possibility of over estimating or under-estimating the nano-hardness value [39]. On the other hand, a complete homogenous dispersion of carbide within the metal matrix in CoCrMo alloy is practically impossible. Hence, it is essential to select an area that best represents the material condition.

## 4. Tribocorrosion Modelling

From the standpoint of applications, it is vital to distinguish the contribution of wear, corrosion, and the synergetic contribution of both components for effective material degradation control. Hence, this section of the review summarizes the three common quantitative approaches in describing the wear-corrosion relationship.

### 4.1. Synergistic Approach

This is by far the simplest and the first approach in quantifying the wear-corrosion interaction. The total material loss due to tribocorrosion, *T*, is given in Equation (4) [8].

(4) T=W+C+S

where, *W* is the material loss due to wear without corrosion, *C* is the material loss due to corrosion only (static conditions), and *S* is the material degradation effect due to the combination of wear and corrosion. 

Despite the simplicity of the equation, the approach suffers some limitations such as the inability to interpret the tribocorrosion phenomena directly and that the current experimental methods do not allow for the quantification of individual contributions listed in the expression.

### 4.2. Mechanistic Approach

First proposed by Uhlig et al. [40], this approach simplified the total wear volume loss, *U_total_* into two main terms as shown in Equation (5):

(5) $$U_{total}=U_{mechanical}+U_{chemcial}$$

where, the term *U_mechanical_* represents the total volume loss due to pure mechanical wear while *U_chemcical_* is associated with the volume loss due to wear-accelerated corrosion.

It can be assumed that the corrosion through the passivated area of the material is negligible and hence, only the wear-accelerated corrosion occurring through the worn area is considered. Practically, this approach is much simpler to quantify experimentally, where *U_chemical_* can be obtained through the incorporation of excess current derived from the potentiostatic test using Faraday’s law as listed in Equation (6):

(6) $$U_{chemical}=\frac{q\times M}{n\times F}$$

where, *q* is the charge moving across the material under sliding wear (C), *M* is the atomic weight of the element from the material that is dissolved (g/mol), *n* is the corresponding dissolution valence and *F* is Faraday’s constant (96,490 C/mol). Consequently, *U_mechanical_* can be attained from the total volume loss measured.

Recently, Cao et al. [9] further defined the model with the incorporation of the conventional Archard law [41] and an effective load assumption in a mixed lubrication regime. The proposed equation in its simplest form is presented as such:

(7) $$V_{total}=k_{m}\left(\frac{k_{o}F_{n}/h_{min}^{1.49}}{H}\right)+k_{c}\frac{Q_{p}Mv_{s}{\left(\frac{k_{o}F_{n}/h_{min}^{1.49}}{H}\right)}^{0.5}}{nF\rho }$$

where, *V_total_* is the normalized total volume loss per unit time (mm^3^/s), *F_n_* is the normal force (N), *h_min_* is the minimum film thickness (nm), *H* is the micro surface hardness in HV, *Q_p_* is the passivation charge density (mC/cm^2^), *V_s_* is the sliding velocity (mm/s), *ρ* is the density of the material (g/cm^3^), and *k_m_*, *k_c_*, and *k_o_* are the proportional factors defined as such *k_mech_* = *k_m_k_o_*/(2.8^1.49^) and *k_chem_* = *k_m_k_o_*^0.5^/(2.8^0.745^) of which *k_mech_* and *k_chem_* are the proportional mechanical and chemical wear factors, respectively. *k_mech_* and *k_chem_* are system specific and need to be derived specially from experiments. 

Further refinement and calibration can be found elsewhere [42]. Quantitatively, in the current research done by Guadalupe et al. [43], the model has also been able to satisfactorily describe the tribocorrosion behavior for high and low carbon CoCrMo alloys (0.02–0.25 wt % C and 0.01 wt % C, respectively).

### 4.3. Third-Body Approach

**Figure 5:**
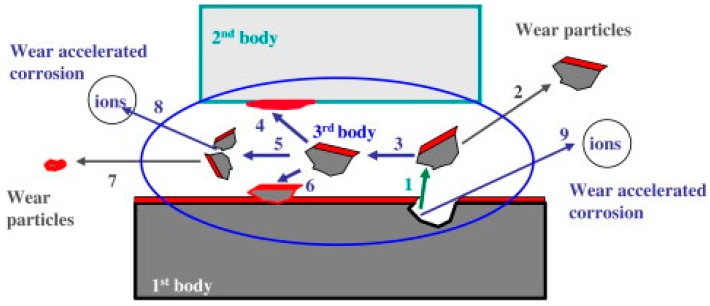
Schematic illustration of material flows and reactions occurring in a tribocorrosion system, with the first body: a passive metal; second body: an inert counter-body and third body: generated from the resulting process. In a tribocorssoion process, the wearing process causes the detachment of the wear particles from the metal (**1**), which would either be ejected (**2**) or transferred to a third body (**3**); Subsequently, the third body can adhere to the counter body (**4**); or fragment into smaller particles (**5**); or the metal (**6**); Upon reaching a critical size, the third body can be ejected from the contact (**7**); In addition, wear accelerated corrosion can be observed in two locations: third body (**8**) and exposed metal (**9**) (occurring immediately after the wear particle detaches) (adapted from reference [21], with permission from © Elsevier).

The last approach aims at addressing the shortfall of the two earlier proposed models. It is presumed by many researchers that different wear mechanisms occur under different wear conditions and these differences are likely to modify the surface chemistry of the materials [23,44]. Accordingly, it would have an effect on the wear-accelerated corrosion and/or corrosion-accelerated wear behavior of the material. Figure 5 shows a schematic illustrating different wear particles and ions that can be ejected from the material surface and their influences on the different wear morphology, *U_mechanical_,* and even *U_chemical_* values of the material [45]. In a study done by Barril et al. [46], it was observed that the trapped particles from the Ti6Al4V alloy loaded against an alumina ball entrapped wear particles that wedged onto the space between the ball and the sample. This in turn affects surface passivation and thus, a higher *U_chemical_* value was observed.

Exploring the third-body approach constituted a hopeful way of explaining the effects of chemical, mechanical, electrochemical, and even the synergistic effects of wear-corrosion phenomena factors on tribocorrosion. Although an explanation is welcome, the complexity in quantitatively interpreting or even predicting the phenomena at the present moment is still theoretically impossible.

## 5. Tribocorrosive Behavior of CoCrMo Alloys

The long-term performance of a CoCrMo alloy depends greatly on the overall tribocorrosion behavior of the material and not just on the individual contribution from wear or corrosion [47]. Hence, it is also important to consider the effects of tribocorrosion on CoCrMo alloys when conditions are varied. In the following sub-sections, we discuss the various parameters related to CoCrMo alloys that supposedly, amongst others, are known to play an influential role in the tribocorrosive behavior of the alloys. 

### 5.1. Metal Carbides

The role of carbides in CoCrMo alloys has been the focus of study in many tribochemical research works. In the specifications of ASTM F75, the standard requires a carbon content of below 0.35 wt %, and there are however, variations in which the alloy can be tailored to suit different needs but still conform to the governing standard. Consequently, CoCrMo alloys can have a high carbon (HC) content (0.05–0.35 wt %) or low carbon (LC) content (<0.05 wt %). It is well known that carbides such as M_23_C_6_ (M = Co., Cr or Mo) and M_6_C are formed during eutectic solidification or precipitation. The transformation of M_23_C_6_ to M_6_C takes place if the CoCrMo alloys are annealed at 1230 °C and above and a complete dissolution of the carbides occurs between 1250 °C and 1270 °C [48]. A low carbon content depresses the solutionizing temperature where a single phase at lower temperatures helps alleviate the problem of incipient melting. It is to be noted that besides the conventional carbides, other phases have also been reported to form in as-cast CoCrMo alloys with varying C content (0.12–0.35 wt %) such as σ phase (Co. (Cr, Mo)) and mixed carbide/nitride phase, π, etc. [49]. However, the exact roles of these phases on tribocorrosion behavior of the alloys are yet to be clearly identified.

In general, the presence of fine carbides helps strengthen the alloys and a better wear resistance is naturally expected [50]. Though not fully understood, it was also experimentally verified by Yan et al. [32] that the presence of carbon increases the surface energy of high carbon CoCrMo alloys compared to low carbon CoCrMo alloys. Consequently, it promotes protein absorption on the alloy surfaces and a decrease in frictional losses. However, various researchers have different opinions on the positive effect of carbides on the wear resistance of CoCrMo alloys. Chiba et al. [51] detailed that LC-forged CoCrMo alloys showed a better wear resistance than the HC-cast CoCrMo alloys mainly due to the fact that the presence of carbides that increase the stacking fault energy thereby preventing SIMT, results in a lower surface fatigue resistance. A similar outcome was also demonstrated by Chen et al. in a recent study between LC- and HC-forged CoCrMo alloys [52].

**Figure 6:**
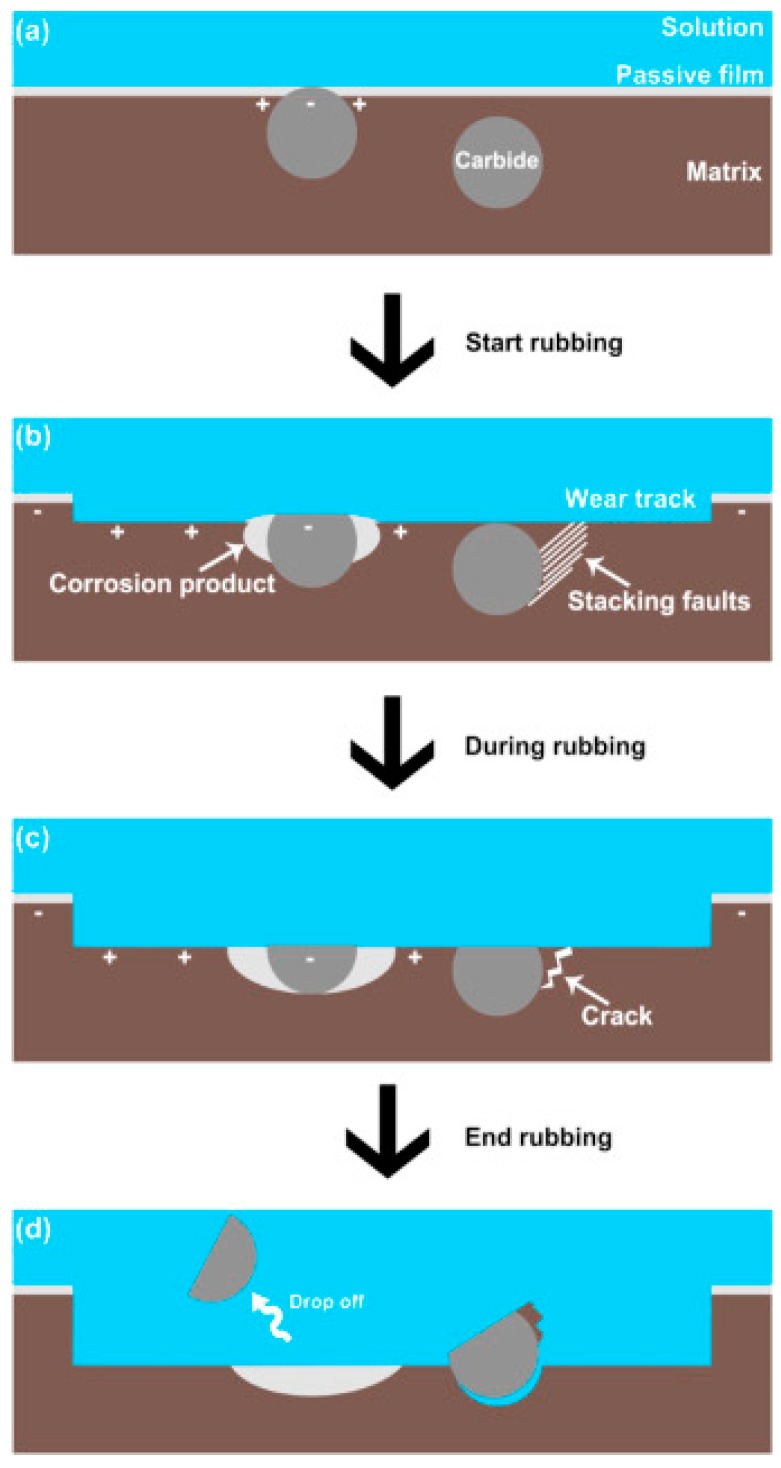
Schematic diagrams showing the proposed tribocorrosion mechanism of HC CoCrMo alloy with time from the start to the end of rubbing (adapted from reference [53], with permission from © Elsevier).

Nevertheless, carbides, no matter how hard they are, would be torn off in the wear process and this may lead to third-body wear. Hence, it is vital to understand how the synergistic effect between wear and corrosion affects the dislodging of carbides from the matrix and inhibits the performance of the CoCrMo alloys. In a recent study by Wang et al. [53], a mechanism was proposed which is schematically shown in Figure 6. It was reported that, under the presence of a biological fluid, a galvanic couple was formed between the carbide and the surrounding matrix, which resulted in a decrease in the Volta potential of the latter. Consequently, the corrosion rate in the region was accelerated with tribocorrosion products that tended to dislodge the carbide particles from the matrix. Additionally, stacking faults were found to accumulate in the phase boundary between the carbide and the matrix, which led to a stress concentration that evolved into surface fatigue in the material in the form of microcracks thereby contributing to the separation of the carbide particles from the matrix. All in all, the dislodged carbide particles could subsequently lead to abrasive wear and low wear resistance. This hypothesis has been accepted in many studies where surface scratches and grooves were observed parallel to the sliding directions [51,54]. Additionally, topological profiling has revealed the carbides to be largely higher than the matrix (~8–9 nm), and these carbide asperities coupled with their higher hardness underwrite the undulations in the friction coefficient curve during sliding, resulting in an overall higher friction coefficient.

**Figure 7:**
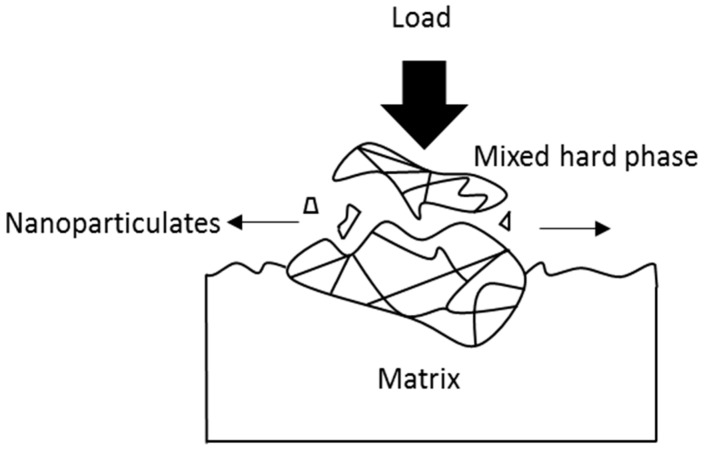
Schematic illustration of nanoparticulates that are generated from the mixed hard phases under a high load.

Nanoparticles generated from tribocorrosion processes have been linked to adverse tissue response inside the human body [55,56]. Such nanoparticles can initiate from either the nanocrystalline surface layer of a CoCrMo alloy in contact with the load or from the agglomerated mixed hard carbide phases (M_23_C_6_ and M_6_C) on the surface that act as obstacles to the path of the abrasive tips [50]. Figure 7 illustrates that carbide/carbide contact under tremendous local stress results in carbide fracture and spalling out from the surface of the alloy [54]. Consequently, this leads to the detachment of the nanograins from the protruded mixed hard phases, thus producing loose bioreactive nanoparticles.

### 5.2. Effects of Alloying Elements

Various alloying elements in elementary CoCr alloys are known to influence the tribocorrosive behavior of the alloys; each alloying element, in general, causes either an alteration of the chemical composition of the parent alloy or results in the formation/inhabitation of phases. The mechanical properties of CoCr alloys have been found to be further enhanced by the additions of tungsten (W) and molybdenum (Mo) [57]. However, the increase in W content tends to promote the formation of intermetallic σ phase and the combination of complex hard phases such as Co_3_Mo/W_3_C, which are detrimental to the fatigue properties of the material in service [50]. Subsequently, the addition of Mo is preferred as it helps improve the ductility of the alloys and promotes the formation of eutectic carbides instead of the complex ones discussed earlier. In an earlier study reported by Shin et al. [58], it was also found that the addition of Mo helped suppress the formation of M_7_C_3_, which is of a coarser nature, resulting in more weight loss during wear testing. In addition, M_7_C_3_ has a trigonal crystal structure that is of higher hardness compared to M_23_C_6_, a face-centered cubic (FCC) structure. Once these hard M_7_C_3_ carbides spall off from the matrix during wear, they would result in more severe abrasive wear and higher material removal rate [59]. Essentially, a higher Mo content caters for more Cr to remain in the solid-solution matrix rather than forming Cr-rich carbides thus promoting the formation of a dense Cr-oxide film that renders an excellent corrosion resistance to the alloy [60]. 

**Figure 8:**
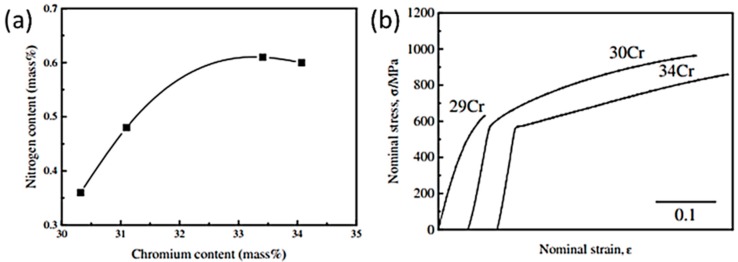
(**a**) The relationship between N and Cr contents and (**b**) the corresponding nominal stress-strain curves of the tested CoCrMo alloys (adapted from reference [63], with permission from © The Japan Institute of Metals and Materials).

It was reported that under as-cast condition, Ni and N free CoCrMo alloys exhibit a low ductility due to the formation of σ phase within the interdendritic region, which results in brittle fracture [61]. Hence, it is vital to suppress the formation of σ phase and, at the same time, stabilize the γ phase through the addition of the fourth element. However, it is also worth noting that although Ni has the same effect as N, it is often undesirable in consideration of the issue of biocompatibility as Ni is a metal sensitizer [62]. Interestingly, in a research done by Lee et al. [63] as summarized in Figure 8, it was revealed that the addition of N to 0.61 wt %, Cr content can be increased to a peak value of 34 wt % (ASTM F75: Cr ≤ 30 wt %), resulting in a significant improvement in mechanical properties compared to the N-free CoCrMo alloys under as-cast condition. The enrichment of Cr content in the alloys is deemed favorable as it enables the formation of Cr-rich oxide film that is essential in biomedical applications.

### 5.3. Effects of Tribocorrosion Conditions

In-vitro and retrieval studies [35] of CoCrMo implants have often revealed tribocorrosion reactions involving the interactions between biological, chemical and mechanical wear [64,65,66,67]. In a biological environment, it is generally accepted that the dominant underlying mechanism for material mass loss is probably due to abrasion. This has been confirmed on the basis of wear scar analysis showing a parallel and uniformly grooved pattern on the wear surface. However, as mentioned previously, the total mass loss is not simply the summation of corrosion wear and mechanical wear losses [68]. There is the so-called ‘synergistic component’ as demonstrated by Mathew et al. [69] in a study investigating the effects of protein on CoCrMo alloys. Their study using bovine serum quantified the individual mass loss contributions and showed that around one-third of the mass loss was due to this synergistic effect. Hence, it is of no surprise that any change in the environment chemistry would affect the tribocorrosive behavior of alloys [70,71,72]. The most commonly investigated solutions include sodium chloride solution (NaCl 0.36%), phosphate buffered solution (PBS), Hank’s solution, and Ringer solution. 

For the purpose of simulating the presence of amino acids in the biological environment, Yan et al. [34] reported an experiment using Dulbecco’s Modified Eagle’s Medium (DMEM) as the lubricating medium in tribocorrosion analysis of a CoCrMo alloy. Remarkably, the results demonstrated reduced coefficient of friction and lower wear rate compared to similar CoCrMo alloys reported elsewhere [32]. Through the findings, the authors drew the conclusion that a reaction existed between the amino acid and the bulk metal and the metal ions resulted in the formation of a thin tribo-film (~2–3 nm), thereby reducing friction and wear. It is also worth noting that in electrochemical tribo-testing, CoCrMo alloys can be further made active by applying an external potential thus affecting the rate of reaction and the formation of tribo-film [73]. A more recent study by Igual-Muñoz et al. [72] revealed a similar finding through experiment using NaCl + albumin. Conversely, the albumin acted as a binder by promoting the agglomeration of suspended hard particles and thus reducing the effect of abrasive wear. Notwithstanding that, the presence of protein may also present unfavorable consequences. In another study done by Yan et al. [56], it was observed that the presence of protein could undeniably accelerate the formation of ions, such implants when used in vivo would have cytotoxic effects on the neighboring cells [74,75].

In a separate study done by Sun et al. [76] representing an infected joint condition, it was found that an acidic environment (pH 4.0) accelerated the release of metal ions and, when coupled with the presence of third-body particles, aggravated material removal, which would impose a health hazard when implanted. On the whole, a general conclusion from the previous studies suggests the pivotal roles of chemical reactions that occur at the metal/electrolyte interfaces. Consequently, it is vital that researchers consider the appropriate surface chemical phenomena in the evaluation of biomedical alloys. 

### 5.4. Influence of Wear Debris

Metal-on-metal (MOM) implants are intended to offer improved durability, releasing a lower volume of wear debris than traditional polymer or ceramic-on-metal (POM/COM) implant designs. However, the implants are known to release particles and soluble wear debris upon mutual contact, which are consumed in the bloodstream. This in turn leads to a permanently increased content of metal ions in the hip synovial fluid and in the peripheral blood stream, which can have severe side effects on the patients subjected to long-term implant usage [77,78,79,80,81]. To further complicate matters, these metal particles may also spread through lymphatic circulation and continue to release ions even after removal of the source of wear [82]. Current evidence indicates that the sizes of wear particles generated by CoCrMo MOM articulations are generally in a nanometer range [83]. A large surface area enhances the release of metal ions, predominantly Co. and Cr ions, into the circulation [84]. However, the preferential release of the metal ions is still largely dependent on the experimental or operational environment.

**Figure 9:**
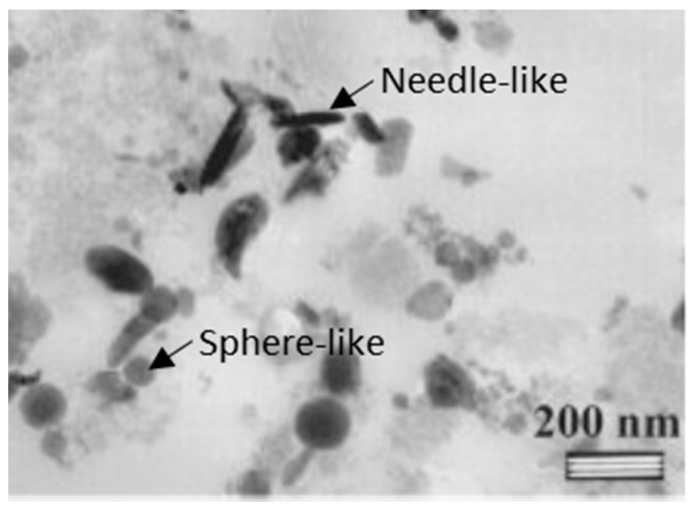
Transmission electron microscopy (TEM) micrograph of different shapes of the wear particles generated from a metal-on-metal (MOM) implant pair of HC-CoCrMo alloy in 95% bovine serum (adapted from reference [85], with permission from © John Wiley and Sons).

Some studies reported that in vitro or in vivo wear particles are mainly chromium (Cr) oxide. In bovine calf serum medium, Catelas et al. [85] reported Cr-oxide to be the majority phase in both HC and LC CoCrMo alloys. Although it was observed that some of the wear debris had a CoCrMo composition with traces of carbides, the predominant wear-inducing particles are still the oxide particles without Co. The shapes of the particles as depicted in Figure 9, range from needle-like to spherical morphology with a size range of 25–50 nm. Contradictorily, Yan et al. [86] tested nanosized wear debris of CoCrMo alloy in a protein containing bovine serum albumin (BSA) medium. It was observed in this study that the crystalline wear debris was comprised of two constituents instead of one, that is Cr oxide and the other being crystalline Co with a close-packed hexagonal (hcp) structure due to the transformation of fcc-Co to hcp-Co under significant plastic deformation during wear [87]. Nevertheless, it is still unclear, which alterations a wear particle goes through between its generation and its detachment in the joint environment. It may be possible that the particles undergo significant transformation and changes in chemical composition within the tribological interface leading to the disruption of the organic protective film and subsequent particle oxidation or perhaps just the spalling off of insoluble Cr-oxide particles due to the passivized film.

### 5.5. Influences of Manufacturing Methods

**Figure 10:**
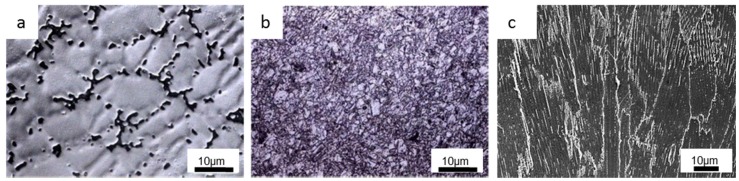
(**a**,**b**) Optical images on the typical microstructure of the (**a**) cast and (**b**) forged CoCrMo alloys (adapted from reference [51], with permission from © Elsevier) and (**c**) SEM image of selective electron beam melting (SEBM)-built CoCrMo alloy.

Very often, different manufacturing process routes have been explored to tailor the CoCrMo alloys’ microstructure and their entailing tribological properties based on practical feasibility. The first generation of CoCrMo implants was produced based on casting [88], which possessed discontinuous, discrete interdendritic, and blocky carbides of mainly M_23_C_6_ within the Co-matrix as depicted in Figure 10a [35,50,51,89]. Owing to the casting process, carbide inhomogeneities related to size and dispersion rendered low ductilities and fatigue strengths [90], which in turn hindered their long-term performance when implanted. Nonetheless, heat treatment can be applied to dissolve the large carbides into the intergranular region and improve the performance of the alloys [91]. On the aspect of tribocorrosion, blocky carbides are deemed unfavorable due to a severe spalling effect that results in more mechanical wear loss of the alloys [92]. On the contrary, as reported by Sun et al. [28], bigger abrasive sizes may instead appear more favorable as smaller-sized abrasive particles could undermine the repassivation effect of the CoCrMo alloys due to shorter recovery time. Consequently, the exposed CoCrMo alloys are subjected to adverse effects arising from wear-induced corrosion.

The forged CoCrMo alloys represented an improvement over the cast alloys and have been extensively employed for use as articulating surfaces of implants [93,94]. This technique utilizes the combined effects of plastic deformation with subsequent recrystallization heat treatment to achieve fine grain sizes that strengthen the alloys and further enhance their wear resistance at the expense of ductility [95]. An optical image on the microstructure of a typical forged CoCrMo alloy is shown in Figure 10b [51]. However, it is suggested in the study reported by Buscher et al. [96,97] that the forging process results in anisotropic property of CoCrMo alloys due to the formation of ε martensite and stacking faults. Subsequently, a nanocrystalline layer is generated just below the surface during wear, which supports the hypothesis that the generation of globular and needle-like particles is due to the torn-off nanocrystals and fractured ε martensite, respectively. In contrast, surface fatigue within the nanocrystalline layer is proposed to be the acting wear mechanism in MOM articulation instead of the proposed abrasive or oxidative wear (tribochemical reaction). Recently, considerable attention has been given to ultrafine-grained (UFG) materials due to their superior strengths that co-exist with good fracture toughness and excellent plasticity [98]. Subsequently, Kenta et al. [15] developed a new strategy to produce high strength CoCrMo alloys with sufficient ductility through dynamic recrystallization (DRX) via hot forging. Through the addition of optimized N content, Kenta and co-workers successfully stabilized the γ phase (fcc-Co.) and prevented both athermal ε martensite and SIMT during deformation. This produced an ultrafine microstructure with grain sizes typically smaller than 1 μm, which caused an enhanced work hardening in the alloys.

The capability of AM technologies has improved tremendously over the last decade. With optimized process parameters, near-fully dense CoCrMo parts can be relatively easily fabricated via various AM techniques such as selective electron beam melting (SEBM), selective laser melting (SLM), laser engineered net shaping (LENS), etc. [99,100,101,102]. The former two techniques being the most popular, are essentially powder bed fusion AM methods that utilizes a heat source (laser/electron beam) to selectively melt and fuse multiple layers of powder together to form the desired object [103,104]. Consequently, SBEM is also a technique that is fast gaining popularity due to its excellent building and energy efficiency [105,106,107,108]. A near vacuum fabrication environment makes it an added advantage especially in the area of bio-related applications. The high thermal gradients coupled with rapid solidification offer a uniquely columnar microstructure along the build direction, with carbide precipitates that appear continuously at the grain boundaries and dispersed as clusters in the interdendritic regions [16,109,110]. An SEM micrograph showing a typical SEBM-built CoCrMo part is depicted in Figure 10c. Interestingly, in a recent study by Tan et al. [111], through extensive characterization, it was suggested that a weak incoherent interface between the grain boundary carbide and one side of the γ-Co. grains is the source of anisotropy in the SEBM-built CoCrMo alloy. Consequently, it is more likely that these nano-scale carbides could be dislodged from the matrix, which would then have a significant effect on the tribocorrosion behavior of the alloy. Hence, it was further suggested that the carbon content in the CoCrMo powders can be made lower to facilitate the production of discrete nano-scale carbides, which would strengthen the materials rather than contribute to third-body abrasives.

## 6. Summary

**Figure 11:**
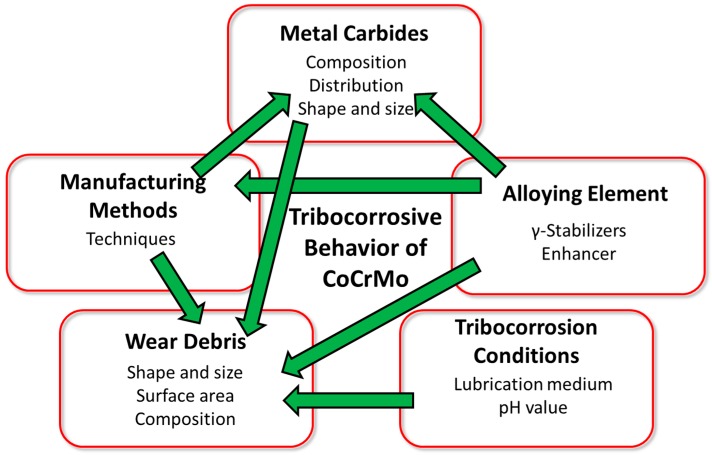
Schematic illustration of the five types of influence affecting the tribocorrosive behavior of CoCrMo alloys.

This article reviews the various methods used in electrochemistry, which have been subsequently employed for use in the evaluation of the tribocorrosive behavior of CoCrMo alloys. In light of this, the commonly used approaches and latest proposed models to describe the tribocorrosive behavior of CoCrMo alloys were reviewed. In addition, an insight on the several factors that affect the tribocorrosion behavior of CoCrMo alloys was also discussed and is portrayed in Figure 11, where the green arrows signify the interactions of the various parameters.

Despite the diverse experimental conditions and procedures reported in recent literature, the following remarks have been drawn:

- Various tribochemical methods are, in general employed to effectively characterize the tribocorrosive behavior of CoCrMo alloys. Although the OCP method can reveal the necessary potential of an alloy before, during, and after wear test, it is unable to provide the state in which the alloy exists. An alloy after wear can indeed recover and then exhibits the same corrosion potential as before wear but an EIS measurement can successfully identify a change in electrical impedance of the alloy that designates an easier route for a subsequent current to flow and a reduced corrosion resistance. In practice, OCP and EIS both render convenient and well-accepted methods to quantitatively evaluate the tribocorrosive properties of the concerned alloy. However, methods such as potentiostatic and cathodic protection should not be ignored as they provide important means of evaluating the tribocorrosion process kinetics occurring between the alloy and the environment during wear.
- A simplistic tribocorrosion model can be further improved into more precise models governing the tribocorrosion process of CoCrMo alloys with the aid of tribochemical approaches. In addition, these tribochemical approaches when coupled with an analytical technique such as inductive coupled plasma mass spectroscopy (ICP-MS), which is often used to detect metal ions and their concentrations, could be utilized to explain some of the phenomena that cannot be fully described by the present models.
- Long-term performance of CoCrMo alloys largely depends on the applied environmental conditions. Carbides play a pivotal role in the strengthening of CoCrMo alloys but also introduce unfavorable tribocorrosion conditions that are detrimental to the materials when dislodged from the matrices. In general, LC-CoCrMo alloys show a better wear resistance than HC-CoCrMo alloys. Alloying elements, such as N, can be added to strengthen the alloys through the formation of nanograins and the use of carbon as a strengthener in CoCrMo alloys is perhaps restricted. Consequently, more Cr ions can be made free to form protective Cr-oxide thereby increasing the tribocorrosive resistance of the alloys. Inevitably, the manufacturing process route of a CoCrMo alloy also plays an important role through variations of microstructure and resulting wear characteristics. Very often, the rule of thumb is to lower the volumetric wear rate. However, it does not always equate to a lower number of emitted nanoparticles. Hence, it is also of equal importance to quantify and characterize the release of these nanoparticles because this will lead to more pronounced release of metal ions due to a surface area effect. Though ion release may constitute negative effects on the surrounding tissues, it must be acknowledged that these ions sometimes can also react with the proteins present in the environment to form a tribofilm that tends to protect the surface from further wear.

Independently, tribo-mechanisms already exhibit high complexities and non-linear behaviors but the introduction of corrosion further complicates the issue. In fact, most of the metal loss occurs due to the synergistic effect of the different mechanisms. Therefore, the understanding of chemical, mechanical, and biological phenomena involved in the tribocorrosion process helps distinguish the different pathways of the degradation process. Ultimately, this knowledge will help mitigate material loss and improve the tribological performance of future CoCrMo alloys, which will have a direct impact on patients’ lives. On the subsequent research outlook, it may also be of interest to further explore and understand the role of single abrasive particles on the nanoscale tribocorrosion of CoCrMo alloys should it be effectively reduced to a nanosized bacteria robot, where targeted drug delivery for cancer or other forms of nanoscale surgery could be realized.

## Figures and Tables

**Table 1:** Chemical compositions (wt %) of Stellite^TM^ 21 and Arcam EBM CoCrMo compared to the chemical requirements of ASTM F75.

Element (wt %)	Co	Cr	Mo	Ni	C	Si
ASTM F75	Bal.	27–30	5–7	<0.50	<0.35	<1.0
Stellite^TM^ 21	Bal.	25–29	5–6	1.75–3.75	0.20–0.30	1.0
Arcam EBM CoCrMo	Bal.	28.5	6	0.25	0.22	0.7

**Table 2:** Average wear volumes obtained for HC CoCrMo alloy in NaCl and bovine serum at 0.05 and 0.5 *V_Ag/AgCl_* (×10^−3^ mm^3^) (data extracted from [31]).

Solution	Applied Potential (*V_Ag/AgCl_*)	*U_chemical_* (mm^3^)	*U_mechanical_* (mm^3^)	*U_total_* (mm^3^)
NaCl	0.05	8.9	11.1	20.0
Bovine serum		7.5	20.9	28.4
NaCl	0.5	17.2	10.0	27.2
Bovine serum		7.9	20.1	28.0

## References

[1] Landolt D., Mischler S. (2011). Tribocorrosion of Passive Metals and Coatings.

[2] Yan Y. (2013). Bio-Tribocorrosion in Biomaterials and Medical Implants.

[3] Zhang S., Zhao D. (2012). Aerospace Materials Handbook.

[4] Yan Y. (2006). Corrosion and Trib-Corrosion Behaviour of Metallic Orthopaedic Implant Materials. Ph.D. Thesis.

[5] Anaee R.A.M., Abdulmajeed M.H. (2016). Tribocorrosion. Advances in Tribology.

[6] Barão V., Sukotjo C., Mathew M. (2013). Fundamentals of Linking Tribology and Corrosion (Tribocorrosion) for Medical Applications: Bio-Tribocorrosion. Tribology for Scientists and Engineers.

[7] Landolt D. (2006). Electrochemical and materials aspects of tribocorrosion systems. J. Phys. D Appl. Phys..

[8] Watson S., Friedersdorf F.J., Madsen B.W., Cramer S.D. (1995). Methods of measuring wear-corrosion synergism. Wear.

[9] Cao S., Maldonado S.G., Mischler S. (2015). Tribocorrosion of passive metals in the mixed lubrication regime: Theoretical model and application to metal-on-metal artificial hip joints. Wear.

[10] Toumey C. (2013). Nanobots Today. Nat. Nanotechnol..

[11] Sitti M. (2009). Miniature devices: Voyage of the microrobots. Nature.

[12] Asgar K., Peyton F.A. (1961). Effect of microstructure on the physical properties of cobalt-base alloys. J. Dent. Res..

[13] Hallab N.J., Jacobs J.J. (2009). Biologic effects of implant debris. Bull. NYU Hosp. Jt. Dis..

[14] Okazaki Y. (2008). Effects of heat treatment and hot forging on microstructure and mechanical properties of Co-Cr-Mo alloy for surgical implants. Mater. Trans..

[15] Yamanaka K., Mori M., Chiba A. (2012). Enhanced mechanical properties of as-forged Co-Cr-Mo-N alloys with ultrafine-grained structures. Metall. Mater. Trans. A.

[16] Toh W.Q., Sun Z., Tan X., Liu E., Tor S.B., Chua C.K. Comparative Study on Tribological Behavior of Ti-6Al-4V and Co-Cr-Mo Samples Additively Manufactured with Electron Beam Melting. Proceedings of the 2nd International Conference on Progress in Additive Manufacturing (Pro-AM 2016).

[17] Landolt D. (2007). Corrosion and Surface Chemistry of Metals.

[18] Mischler S., Debaud S., Landolt D. (1998). Wear-accelerated corrosion of passive metals in tribocorrosion systems. J. Electrochem. Soc..

[19] Amstutz H.C., Campbell P., McKellop H., Schmalzried T.P., Gillespie W.J., Howie D., Jacobs J., Medley J., Merritt K. (1996). Metal on metal total hip replacement workshop consensus document. Clin. Orthop. Relat. Res..

[20] Savarino L., Granchi D., Ciapetti G., Cenni E., Nardi Pantoli A., Rotini R., Veronesi C.A., Baldini N., Giunti A. (2002). Ion release in patients with metal-on-metal hip bearings in total joint replacement: A comparison with metal-on-polyethylene bearings. J. Biomed. Mater. Res. Part A.

[21] Mischler S. (2008). Triboelectrochemical techniques and interpretation methods in tribocorrosion: A comparative evaluation. Tribol. Int..

[22] Mischler S., Spiegel A., Stemp M., Landolt D. (2001). Influence of passivity on the tribocorrosion of carbon steel in aqueous solutions. Wear.

[23] Landolt D., Mischler S., Stemp M. (2001). Electrochemical methods in tribocorrosion: A critical appraisal. Electrochim. Acta.

[24] Ponthiaux P., Wenger F., Drees D., Celis J.P. (2004). Electrochemical techniques for studying tribocorrosion processes. Wear.

[25] Goldberg J.R., Gilbert J.L. (1997). Electrochemical response of CoCrMo to high-speed fracture of its metal oxide using an electrochemical scratch test method. J. Biomed. Mater. Res..

[26] Kim J.-D., Pyun S.-I. (1996). The effects of applied potential and chloride ion on the repassivation kinetics of pure iron. Corros. Sci..

[27] Jemmely P., Mischler S., Landolt D. (2000). Electrochemical modeling of passivation phenomena in tribocorrosion. Wear.

[28] Sun D., Wharton J., Wood R. (2009). Abrasive size and concentration effects on the tribo-corrosion of cast CoCrMo alloy in simulated body fluids. Tribol. Int..

[29] Fricke H. (1932). XXXIII. The theory of electrolytic polarization. Lond. Edinb. Dublin Philos. Mag. J. Sci..

[30] Azzi M., Szpunar J. (2007). Tribo-electrochemical technique for studying tribocorrosion behavior of biomaterials. Biomol. Eng..

[31] Munoz A.I., Julián L.C. (2010). Influence of electrochemical potential on the tribocorrosion behaviour of high carbon CoCrMo biomedical alloy in simulated body fluids by electrochemical impedance spectroscopy. Electrochim. Acta.

[32] Yan Y., Neville A., Dowson D., Williams S. (2006). Tribocorrosion in implants—Assessing high carbon and low carbon Co–Cr–Mo alloys by in situ electrochemical measurements. Tribol. Int..

[33] Von Baeckmann W., Schwenk W., Prinz W. (1997). Handbook of Cathodic Corrosion Protection.

[34] Yan Y., Neville A., Dowson D. (2007). Tribo-corrosion properties of cobalt-based medical implant alloys in simulated biological environments. Wear.

[35] Liao Y., Hoffman E., Wimmer M., Fischer A., Jacobs J., Marks L. (2013). CoCrMo metal-on-metal hip replacements. Phys. Chem. Chem. Phys..

[36] Pourbaix M. (1974). Atlas of Electrochemical Equilibria in Aqueous Solutions.

[37] Sun D., Wharton J., Wood R. (2011). Micro-and nano-scale tribo-corrosion of cast CoCrMo. Tribol. Lett..

[38] Martinez Nogues V. (2016). Nano-Scale Tribocorrosion of CoCrMo Biomedical Alloys. Ph.D. Thesis.

[39] Maruyama N., Kawasaki H., Yamamoto A., Hiromoto S., Imai H., Hanawa T. (2005). Friction-wear properties of nickel-free Co–Cr–Mo alloy in a simulated body fluid. Mater. Trans..

[40] Uhlig H.H. (1954). Mechanism of fretting corrosion. J. Appl. Mech. Trans. ASME.

[41] Archard J. (1953). Contact and Rubbing of Flat Surfaces. J. Appl. Phys..

[42] Maldonado S.G., Mischler S., Cantoni M., Chitty W.J., Falcand C., Hertz D. (2013). Mechanical and chemical mechanisms in the tribocorrosion of a Stellite type alloy. Wear.

[43] Guadalupe S., Cao S., Cantoni M., Chitty W.J., Falcand C., Mischler S. (2017). Applicability of a recently proposed tribocorrosion model to CoCr alloys with different carbides content. Wear.

[44] Godet M. (1984). The third-body approach: A mechanical view of wear. Wear.

[45] Landolt D., Mischler S., Stemp M., Barril S. (2004). Third Body Effects and Material Fluxes in Tribocorrosion Systems Involving a Sliding Contact. Wear.

[46] Barril S., Mischler S., Landolt D. (2005). Electrochemical effects on the fretting corrosion behaviour of Ti6Al4V in 0.9% sodium chloride solution. Wear.

[47] Mischler S., Munoz A.I. (2013). Wear of CoCrMo alloys used in metal-on-metal hip joints: A tribocorrosion appraisal. Wear.

[48] Clemow A., Daniell B. (1979). Solution treatment behavior of Co-Cr-Mo alloy. J. Biomed. Mater. Res. Part A.

[49] Mineta S., Namba S., Yoneda T., Ueda K., Narushima T. (2010). Carbide formation and dissolution in biomedical Co-Cr-Mo alloys with different carbon contents during solution treatment. Metall. Mater. Trans. A.

[50] Liao Y., Pourzal R., Stemmer P., Wimmer M.A., Jacobs J.J., Fischer A., Marks L.D. (2012). New insights into hard phases of CoCrMo metal-on-metal hip replacements. J. Mech. Behav. Biomed. Mater..

[51] Chiba A., Kumagai K., Nomura N., Miyakawa S. (2007). Pin-on-disk wear behavior in a like-on-like configuration in a biological environment of high carbon cast and low carbon forged Co–29Cr–6Mo alloys. Acta Mater..

[52] Chen Y., Li Y., Kurosu S., Yamanaka K., Tang N., Koizumi Y., Chiba A. (2014). Effects of sigma phase and carbide on the wear behavior of CoCrMo alloys in Hanks’ solution. Wear.

[53] Wang Z., Yan Y., Xing L., Su Y., Qiao L. (2017). The role of hard phase carbides in tribocorrosion processes for a Co-based biomedical alloy. Tribol. Int..

[54] Wimmer M., Loos J., Nassutt R., Heitkemper M., Fischer A. (2001). The acting wear mechanisms on metal-on-metal hip joint bearings: In vitro results. Wear.

[55] Behl B., Papageorgiou I., Brown C., Hall R., Tipper J.L., Fisher J., Ingham E. (2013). Biological effects of cobalt-chromium nanoparticles and ions on dural fibroblasts and dural epithelial cells. Biomaterials.

[56] Yan Y., Neville A., Dowson D., Williams S., Fisher J. (2009). Effect of metallic nanoparticles on the biotribocorrosion behaviour of Metal-on-Metal hip prostheses. Wear.

[57] Al Jabbari Y.S. (2014). Physico-mechanical properties and prosthodontic applications of Co-Cr dental alloys: A review of the literature. J. Adv. Prosthodont..

[58] Shin J.-C., Doh J.M., Yoon J.K., Lee D.Y., Kim J.S. (2003). Effect of molybdenum on the microstructure and wear resistance of cobalt-base Stellite hardfacing alloys. Surf. Coat. Technol..

[59] Niinomi M. (2008). Metallic biomaterials. J. Artif. Organs.

[60] Lin C.-Y., Chang C.-H., Tsai W.-T. (2004). Morphological and microstructural aspects of metal dusting on 304L stainless steel with different surface treatments. Oxid. Met..

[61] Sukaryo S.G., Purnama A., Hermawan H. (2016). Structure and Properties of Biomaterials. Biomaterials and Medical Devices.

[62] Nicholson J.W. (2007). The Chemistry of Medical and Dental Materials.

[63] Lee S.-H., Nomura N., Chiba A. (2008). Significant improvement in mechanical properties of biomedical Co-Cr-Mo alloys with combination of N addition and Cr-enrichment. Mater. Trans..

[64] Batchelor A.W., Loh N.L., Chandrasekaran M. (2011). Materials Degradation and Its Control by Surface Engineering.

[65] Vidal C.V., Muñoz A.I. (2011). Effect of physico-chemical properties of simulated body fluids on the electrochemical behaviour of CoCrMo alloy. Electrochim. Acta.

[66] Yan Y., Neville A., Dowson D. (2006). Biotribocorrosion—An appraisal of the time dependence of wear and corrosion interactions: I. The role of corrosion. J. Phys. D Appl. Phys..

[67] Yan Y., Neville A., Dowson D. (2006). Biotribocorrosion—An appraisal of the time dependence of wear and corrosion interactions: II. Surface analysis. J. Phys. D Appl. Phys..

[68] Sadiq K., Black R.A., Stack M. (2014). Bio-tribocorrosion mechanisms in orthopaedic devices: Mapping the micro-abrasion–corrosion behaviour of a simulated CoCrMo hip replacement in calf serum solution. Wear.

[69] Mathew M.T., Jacobs J.J., Wimmer M.A. (2012). Wear-corrosion synergism in a CoCrMo hip bearing alloy is influenced by proteins. Clin. Orthop. Relat. Res..

[70] Sun D., Wharton J.A., Wood R.J.K., Ma L., Rainforth W.M. (2009). Microabrasion–corrosion of cast CoCrMo alloy in simulated body fluids. Tribol. Int..

[71] Wang Z., Yan Y., Su Y., Qiao L. (2016). Effect of proteins on the surface microstructure evolution of a CoCrMo alloy in bio-tribocorrosion processes. Colloids Surf. B Biointerfaces.

[72] Muñoz A.I., Mischler S. (2011). Effect of the environment on wear ranking and corrosion of biomedical CoCrMo alloys. J. Mater. Sci. Mater. Med..

[73] Stojadinović J., Bouvet D., Declercq M., Mischler S. (2009). Effect of electrode potential on the tribocorrosion of tungsten. Tribol. Int..

[74] Posada O.M., Tate R.J., Meek R.M., Grant M.H. (2015). In vitro analyses of the toxicity, immunological, and gene expression effects of cobalt-chromium alloy wear debris and Co ions derived from metal-on-metal hip implants. Lubricants.

[75] Kanaji A., Orhue V., Caicedo M.S., Virdi A.S., Sumner D.R., Hallab N.J., Yoshiaki T., Sena K. (2014). Cytotoxic effects of cobalt and nickel ions on osteocytes in vitro. J. Orthop. Surg. Res..

[76] Sun D., Wharton J., Wood R. (2008). Effects of proteins and pH on tribocorrosion performance of cast CoCrMo—A combined electrochemical and tribological study. Tribol. Mater. Surf. Interfaces.

[77] D’Adda F., Borleri D., Migliori M., Mosconi G., Medolago G., Virotta G., Colombo F., Seghizzi P. (1994). Cardiac function study in hard metal workers. Sci. Total Environ..

[78] Seghizzi P., D’adda F., Borleri D., Barbic F., Mosconi G. (1994). Cobalt myocardiopathy. A critical review of literature. Sci. Total Environ..

[79] Gilbert C.J., Cheung A., Butany J., Zywiel M.G., Syed K., McDonald M., Wong F., Overgaard C. (2013). Hip pain and heart failure: The missing link. Can. J. Cardiol..

[80] Oldenburg M., Wegner R., Baur X. (2009). Severe cobalt intoxication due to prosthesis wear in repeated total hip arthroplasty. J. Arthroplast..

[81] Tower S.S. (2010). Arthroprosthetic cobaltism: Neurological and cardiac manifestations in two patients with metal-on-metal arthroplasty. JBJS Case Connect..

[82] Hiromoto S., Onodera E., Chiba A., Asami K., Hanawa T. (2005). Microstructure and corrosion behaviour in biological environments of the new forged low-Ni Co–Cr–Mo alloys. Biomaterials.

[83] Hosman A.H., van der Mei H.C., Bulstra S.K., Busscher H.J., Neut D. (2010). Effects of metal-on-metal wear on the host immune system and infection in hip arthroplasty. Acta Orthop..

[84] Lucarelli M., Gatti A.M., Savarino G., Quattroni P., Martinelli L., Monari E., Boraschi D. (2004). Innate defence functions of macrophages can be biased by nano-sized ceramic and metallic particles. Eur. Cytokine Netw..

[85] Catelas I., Bobyn J.D., Medley J.B., Krygier J.J., Zukor D.J., Huk O.L. (2003). Size, shape, and composition of wear particles from metal–metal hip simulator testing: Effects of alloy and number of loading cycles. J. Biomed. Mater. Res. Part A.

[86] Wang Y., Yan Y., Su Y., Qiao L. (2017). Release of metal ions from nano CoCrMo wear debris generated from tribo-corrosion processes in artificial hip implants. J. Mech. Behav. Biomed. Mater..

[87] Pourzal R., Catelas I., Theissmann R., Kaddick C., Fischer A. (2011). Characterization of wear particles generated from CoCrMo alloy under sliding wear conditions. Wear.

[88] Stemmer P., Pourzal R., Liao Y., Marks L., Morlock M., Jacobs J., Wimmer M., Fischer A. (2013). Microstructure of Retrievals Made from Standard Cast HC-CoCrMo Alloys. Metal-On-Metal Total Hip Replacement Devices.

[89] Bettini E., Eriksson T., Boström M., Leygraf C., Pan J. (2011). Influence of metal carbides on dissolution behavior of biomedical CoCrMo alloy: SEM, TEM and AFM studies. Electrochim. Acta.

[90] Hodgson A.W., Virtanen S., Wabusseg H., Totten G.E., Liang H. (2004). Biocompatible metals and alloys: Properties and degradation phenomena in biological environments. Mechanical Tribology Materials, Characterization and Applications.

[91] Varano R., Bobyn J.D., Medley J.B., Yue S. (2006). The effect of microstructure on the wear of cobalt-based alloys used in metal-on-metal hip implants. Proc. Inst. Mech. Eng. Part H J. Eng. Med..

[92] Cao H., Dong X.P., Pan Z., Wu X.W., Huang Q.W., Pei Y.T. (2016). Surface alloying of high-vanadium high-speed steel on ductile iron using plasma transferred arc technique: Microstructure and wear properties. Mater. Des..

[93] Semlitsch M., Willert H. (1980). Properties of implant alloys for artificial hip joints. Med. Biol. Eng. Comput..

[94] Kesh A., Kummer F. New manufacturing and processing techniques for the fabrication of cobalt-chromium surgical implants. Proceedings of the Transactions of 24th Annual Meeting of the Orthopaedic Research Society.

[95] Hermawan H., Ramdan D., Djuansjah J.R. (2011). Metals for biomedical applications. Biomedical Engineering-from Theory to Applications.

[96] Büscher R., Täger G., Dudzinski W., Gleising B., Wimmer M.A., Fischer A. (2005). Subsurface microstructure of metal-on-metal hip joints and its relationship to wear particle generation. J. Biomed. Mater. Res. Part B Appl. Biomater..

[97] Buscher R., Gleising B., Dudzinski W., Fischer A. (2005). Transmission electron microscopy examinations on explanted metal-on-metal hip joints. Prakt. Metall. Pract. Metall..

[98] Ma E. (2003). Instabilities and ductility of nanocrystalline and ultrafine-grained metals. Scr. Mater..

[99] Gaytan S., Murr L.E., Martinez E., Martinez J.L., Machado B.I., Ramirez D.A., Medina F., Collins S., Wicker R.B. (2010). Comparison of microstructures and mechanical properties for solid and mesh cobalt-base alloy prototypes fabricated by electron beam melting. Metall. Mater. Trans. A.

[100] Takaichi A., Nakamoto T., Joko N., Nomura N., Tsutsumi Y., Migita S., Doi H., Kurosu S., Chiba A., Wakabayashi N., Igarashi Y. (2013). Microstructures and mechanical properties of Co–29Cr–6Mo alloy fabricated by selective laser melting process for dental applications. J. Mech. Behav. Biomed. Mater..

[101] Qian B., Saeidi K., Kvetková L., Lofaj F., Xiao C., Shen Z. (2015). Defects-tolerant Co-Cr-Mo dental alloys prepared by selective laser melting. Dent. Mater..

[102] Ram G.J., Esplin C., Stucker B. (2008). Microstructure and wear properties of LENS^®^ deposited medical grade CoCrMo. J. Mater. Sci. Mater. Med..

[103] Frazier W.E. (2014). Metal additive manufacturing: A review. J. Mater. Eng. Perform..

[104] Chua C.K., Leong K.F. (2015). 3D printing and Additive Manufacturing: Principles and Applications.

[105] Toh W.Q., Wang P., Tan X., Nai M.L.S., Liu E., Tor S.B. (2016). Microstructure and Wear Properties of Electron Beam Melted Ti-6Al-4V Parts: A Comparison Study against As-Cast Form. Metals.

[106] Kok Y., Tan X., Tor S.B., Chua C.K. (2015). Fabrication and microstructural characterisation of additive manufactured Ti-6Al-4V parts by electron beam melting. Virtual Phys. Prototyp..

[107] Tan X., Kok Y., Tan Y.J., Descoins M., Mangelinck D., Tor S.B., Leong K.F., Chua C.K. (2015). Graded microstructure and mechanical properties of additive manufactured Ti–6Al–4V via electron beam melting. Acta Mater..

[108] Tan X., Kok Y., Tan Y.J., Vastola G., Pei Q.X., Zhang G., Zhang Y.W., Tor S.B., Leong K.F., Chua C.K. (2015). An experimental and simulation study on build thickness dependent microstructure for electron beam melted Ti–6Al–4V. J. Alloys Compd..

[109] Bordin A., Ghiotti A., Bruschi S., Facchini L., Bucciotti F. (2014). Machinability characteristics of wrought and EBM CoCrMo alloys. Procedia CIRP.

[110] Kircher R., Christensen A., Wurth K. (2009). Electron Beam Melted (EBM) Co-Cr-Mo Alloy for Orthopaedic Implant Applications. Solid Freeform Fabrication Proceedings.

[111] Tan X.P., Wang P., Kok Y., Toh W.Q., Sun Z., Nai M.L.S., Descoins M., Mangelinck D., Liu E., Tor S.B. (2017). Carbide precipitation characteristics in additive manufacturing of Co-Cr-Mo alloy via selective election beam melting. Scr. Mater..

